# Contrast-Enhanced Ultrasound in Residual Tumor of Hepatocellular Carcinoma following Transarterial Chemoembolization: Is It Helpful for Tumor Response?

**DOI:** 10.1155/2018/8632069

**Published:** 2018-08-02

**Authors:** Tian'an Jiang, Qiyu Zhao, Min Huang, Junhui Sun, Guo Tian

**Affiliations:** ^1^Department of Ultrasound, The First Affiliated Hospital, Medical College, Zhejiang University, Hangzhou, China; ^2^Key Laboratory of Precision Diagnosis and Treatment for Hepatobiliary and Pancreatic Tumor of Zhejiang Province, Hangzhou 310003, China; ^3^Hepatobiliary & Pancreatic Intervention Center, The First Affiliated Hospital, Medical College, Zhejiang University, Hangzhou, China

## Abstract

**Aim:**

To investigate the enhancement pattern of residual tumor on contrast-enhanced ultrasonography (CEUS) in patients with hepatocellular carcinoma (HCC) treated with transarterial chemoembolization (TACE).

**Methods:**

Our study initially included 76 patients with HCC, 73 of which were finally allocated into two groups: group 1 (43 patients, post-TACE group) and group 2 (30 patients, untreated HCC group). All patients were performed with CEUS using SonoVue, and qualitative and quantitative enhancement characteristics (rise time, peak time, and washout time) were evaluated for the residual tumors. T test or *χ*2 test was used to estimate for differences between two groups.

**Results:**

In group 1, the mean rise time, peak time, and washout times in group 1 were 16.1±2.7 sec, 31.3±3.1 sec, and 191.0±31.3 sec, respectively. In group 2, these were 15.1±3.5 sec, 30.9±3.2 sec, and 142.6±16.1 sec, respectively. The differences in rise time and peak time were not statistically significant (*P*=0.09 and 0.30, respectively), but the washout time was significantly prolonged in group 1 (*P*<0.01). The enhanced pattern in arterial phase was inhomogeneous (n=11), regular homogeneous (n=11), partial (n=12), peripheral (n=7), and peripheral rim-like (n=2) in group 1. The average of the longest tumor size of the whole lesion in the 5 types was 4.7±1.3cm, 2.9±1.0cm, 3.1±1.7cm, 2.5±0.6cm, and 2.1 cm.

**Conclusion:**

It suggested that the washout time of post-TACE residual lesions was prolonged compared with untreated HCC nodules on CEUS imaging. Combined with the triple-phase enhancement pattern seen on CEUS, the washout time may provide additional information to guide further treatment for residual tumors.

## 1. Introduction

Hepatocellular carcinoma (HCC) is one of the most common primary malignancy tumors throughout the world. However, surgical resection is limited by advanced tumor stage in more than 70% cases [1]. With the widespread adoption of nonsurgical treatments, such as radiofrequency ablation (RFA), percutaneous ethanol injection (PEI), and transarterial chemoembolization (TACE), the need for accurate and noninvasive anatomic and functional assessments of these tumors has increased.

The accurate evaluation of tumor response to nonsurgical treatment is essential for further treatment planning. Angiography, CEUS, CT, and MRI are all considered effective methods for evaluating residual tumors after TACE [2–5]. As for Lipiodol-based TACE, MRI is sensitive for tiny residual tumor and CT is useful for the assessment of iodized oil accumulation. We have achieved reliable results with the combination of various sequences in MR imaging, but the signal of some hepatocellular carcinoma lesions changes from hyperintense to hypointense at short- or long-term follow-up after TACE which can complicate the assessment of these lesions [6]. One pitfall of CT imaging is that accumulation of iodized oil can mask the high-intensity signal of the residual tumor. Arteriography is not suitable for the routine follow-up of treated HCC because of its invasiveness and its limited sensitivity and also is hard to detect small-tumors staining. In the last decade, the use of CEUS for post-TACE lesion assessment has gradually increased. CEUS has been shown to detect residual tumor with a sensitivity from 87% to 100% and a specificity from 65% to 100% and is regarded by some to be superior to CT [7]. Rossa et al. have reported that image fusion of CEUS with CT or MRI improved visualization of microcirculation and residual tumor after TACE, which could increase diagnostic confidence in difficult cases [8]. However, some studies suggest that CEUS has limitations for post-TACE assessment. For example, multiple or deep-seated lesions were difficult to evaluate [3]. In our study, we routinely used CEUS for the study of residual tumors after the first session of TACE, so as to compare the difference between post-TACE and untreated HCC groups.

## 2. Materials and Methods

### 2.1. Study Protocol

This study was a retrospective assessment of 76 consecutive patients with HCC between November 2013 and January 2018, which was registered in Clinicaltrials.gov ID: NCT03026452. In this study, 3 patients were eliminated due to identification by follow-up of MRI examinations, and we finally only included 73 cases proven by pathological examination. In group 1, 43 patients have received TACE treatment. For the control group, we chose another 30 patients with untreated HCC lesions from the same patient database as group 2. All patients gave full informed consent for our study, and the study protocol was approved by our Institutional Review Board.

### 2.2. CEUS Imaging

All CEUS examinations were performed by two skilled investigators with more than 5 years' experience of CEUS. The ultrasound contrast agent (UCA) used in our study was SonoVue® (Bracco, Milan, Italy). The lyophilized powdered SonoVue was reconstituted with the addition of 5 mL of 0.9% sodium chloride, then a dose of 2.4 ml SonoVue was injected through the antecubital vein. All patients underwent the CEUS examination within one month after TACE. All data were acquired using an Esaote MyLab Twice device with CA541 linear probe.

Two investigators (reader 1 who had 8 years of experience and reader 2 who had 20 years of experience) reviewed the imaging and calculated the descriptive statistics in a consensus. The perfusion pattern of each tumor on CEUS during the arterial, the portal, and the late phase was evaluated and classified as hyperenhanced, iso-enhanced, and hypoenhanced. The rise time, peak time, and washout time were determined for the residual tumors. Moreover, in group 1, the enhancement patterns of residual tumors in the arterial phase were classified into five types: inhomogeneous (nodular, branching); homogeneous; partial; peripheral; and peripheral rim-like. Post-Sonovue imaging was divided into three phases: the arterial phase (from 0 to 35 sec), the portal phase (from 35-120 sec), and the late phase (from 120 sec to 5 min). Washout time was defined as the time from contrast appearance to the point where more than eighty percent of the signal disappeared from the lesions.

### 2.3. TACE Procedure

All the 43 patients in group 1 were treated using TACE as the initial therapy. Before TACE, hepatic angiography was performed. Based on information about the feeding vessels, the location of the tumor, and the patients' overall clinical situation, an emulsion of iodized oil (10-50 mL) and chemotherapy drugs of cisplatin (30-40 mg) and fluorouracil (0.5-1.0 g) were injected via the catheter, followed by the injection of a gelatin sponge. The volume of these materials was determined by tumor size.

### 2.4. Statistical Analysis

All data from CEUS imaging in this study was analysed using CnTI software. Quantitative data were expressed as the mean ± SD. T test or *χ*^2^ test was used to estimate for differences between two groups. The value of* p*<0.05 was considered statistically significant. Statistical analyses were performed with the SPSS 16.0 software (SPSS Inc., Chicago, IL, USA).

## 3. Results

The characteristics of patients in two groups are detailed in Table 1. A total of 73 patients were enrolled in this study, including 40 men and 3 women in group 1 and 27 men and 3 women in group 2. There were no significant differences in the ages of two groups (group 1: 56.1±7.9 years; group 2: 49.9±1.1 years) (*p*>0.05), as well as the longest diameter of these tumors which ranged from 1.6 to 7.1 cm (mean: 3.56±1.0 cm) in group 1 and 1.0-8.3 cm (mean: 2.8±0.3 cm) in group 2(*p*>0.05). Furthermore, 34 patients (79.1%) in group 1 and 20 patients (66.7%) in group 2 had single HCC nodules. In the rest of multimodal HCC cases, we chose one proper lesion for the study (n=4 in group 1 and n=5 in group 2). The perfusion characteristics of two groups are described in Table 2.

### 3.1. Quantitative Enhancement Parameters

In group 1, washout time ranged from 40.8 to 300.0 sec (mean: 191.0±31.3 sec). In group 2, washout time ranged from 48.1 to 222.1 sec (mean: 142.6±16.1 sec). There was a significant increase in washout time caused by TACE in group 1 (*p *<0.01). After TACE, 27 residual lesions (62.8%) showed complete washout more than 4 min after the ejection of SonoVue, while the remainder of 16 lesions (37.2%) washed out within 4 min after injection. 25 lesions (83.3%) in group 2 washed out in 4 min.

### 3.2. Arterial Phase

**Figure 1:**
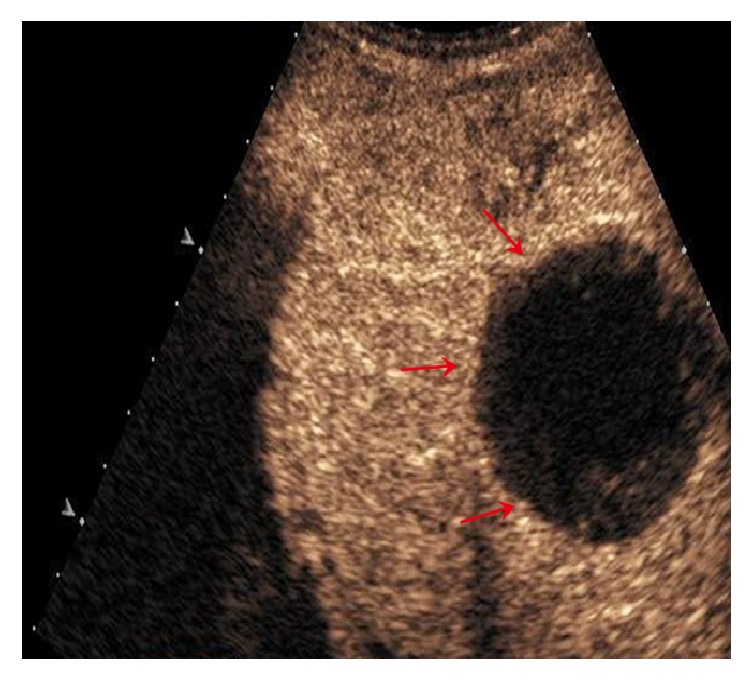
CEUS in fifty-year-old man with complete lesion necrosis (red arrows) after TACE.

**Figure 2:**
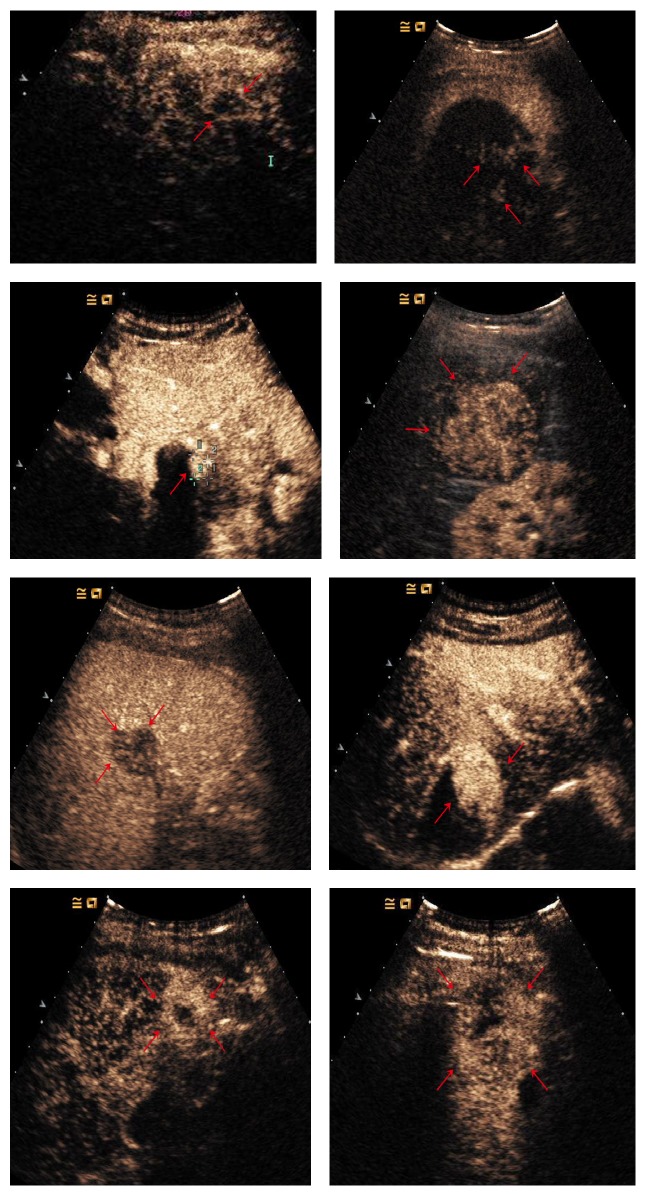
Enhancement patterns in post-TACE group: (a) peripheral rim-like (red arrows); (b, c) inhomogeneous (nodular, branching, etc.) (red arrows); (d, e) homogeneous (red arrows); (f) partial (red arrows); (g, h) peripheral (red arrows).

**Figure 3:**
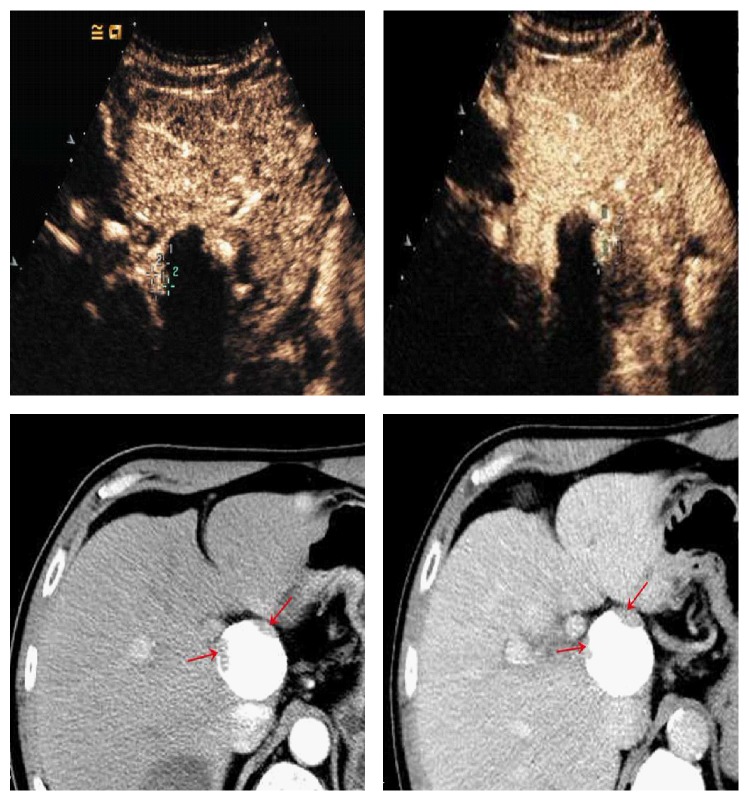
53-year-old man with 4.8cm×4.0 cm tumor. Nodular enhancement (cross mark) seen on CEUS (a, b) was consistent with the contrast-enhanced CT images (c, d) (red arrows).

**Figure 4:**
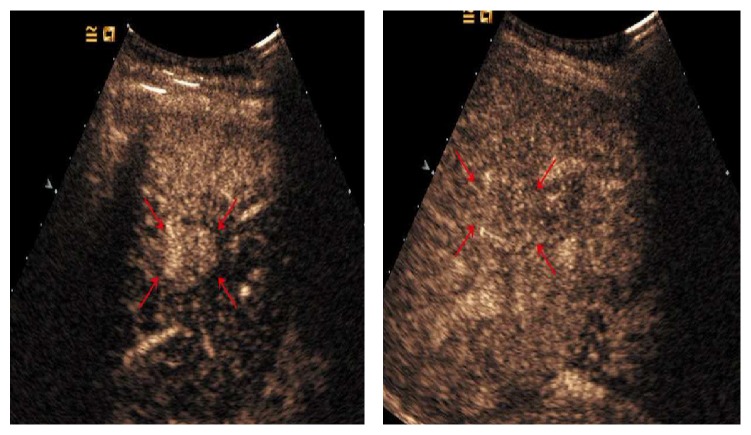
62-year-old man with 2.5 cm×2.1 cm HCC. The lesion shows iso-enhanced in the arterial phase (20.3 sec) (a) (red arrows) and remains iso-enhanced in the portal venous phase (2 min 11 sec) (b) (red arrows).

**Figure 5:**
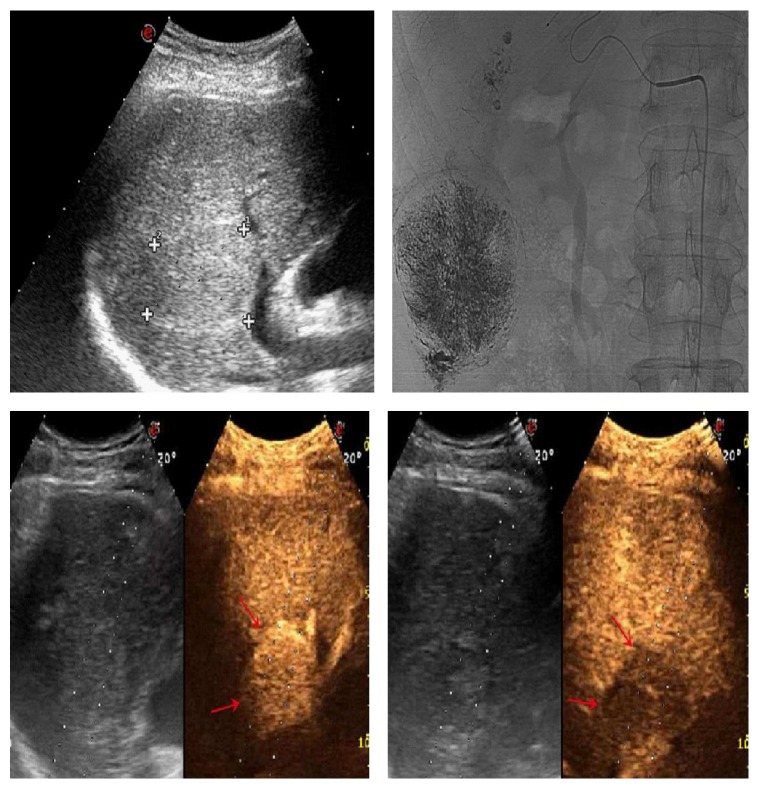
60-year-old man with 4.4 cm×4.2 cm tumor (a) (cross mark). Angiography finding prior to the second session of TACE revealed extensive residual tumor (b). CEUS showed hyperenhancement of the lesion in the arterial phase (c) (red arrows) and washout in the portal venous phase portal venous phase (d) (red arrows).

**Figure 6:**
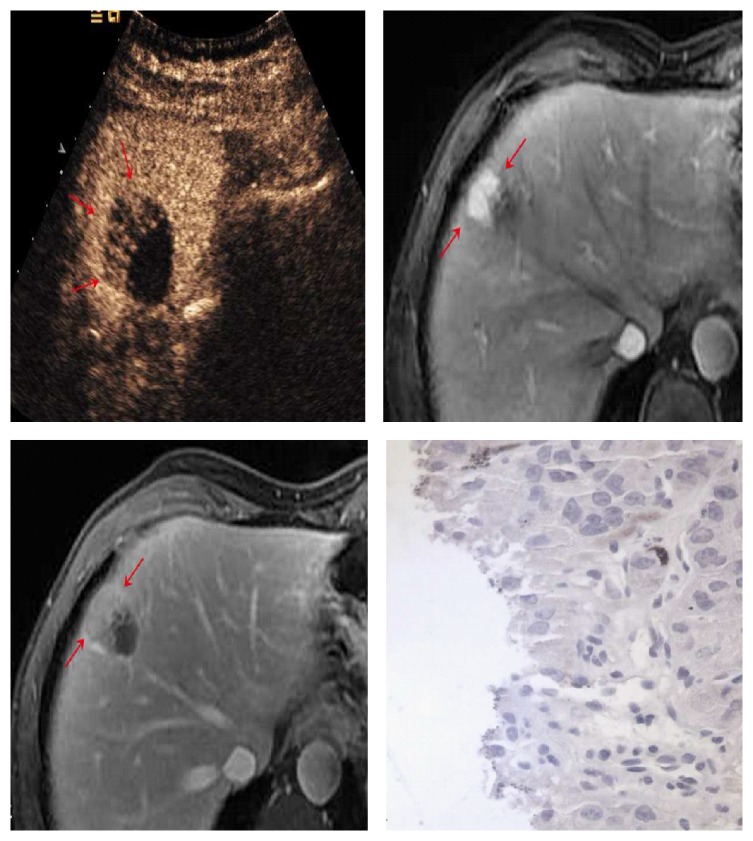
40-year-old man with 3.3 cm×3.2 cm HCC nodule. In portal venous phase, the portal venous phase, the partial enhancement seen at CEUS washed out gradually (a) (red arrows). MR images showed arterial phase enhancement with progressive washout in late phase imaging (b, c) (red arrows). Pathological results showed residual hepatocytes (d).

**Figure 7:**
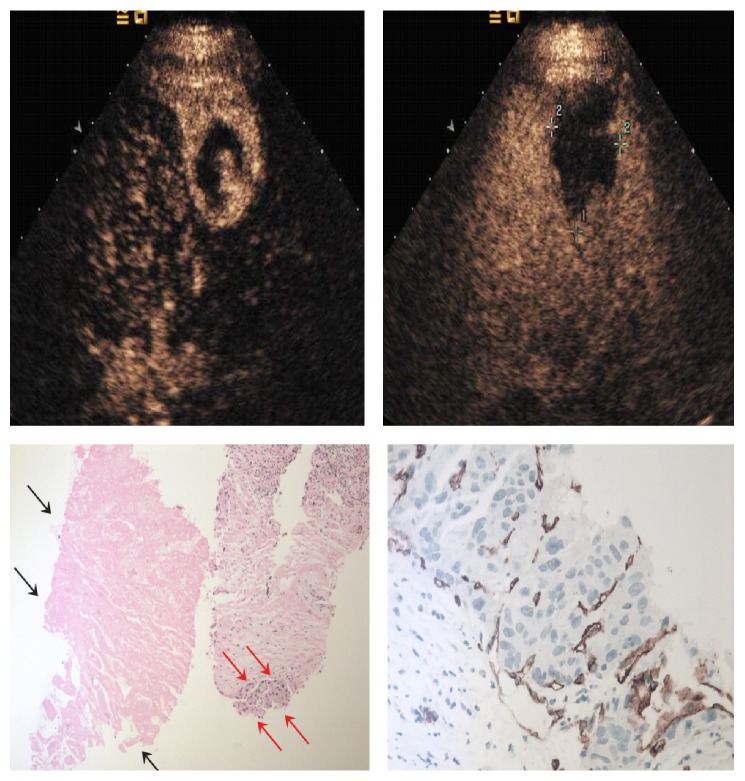
36-year-old man with 4.0 cm×2.8 cm lesion. CEUS images in this large tumor showed peripheral enhancement in the arterial phase (a) and washout in the portal venous phase (b) (cross mark). Percutaneous needle-biopsy results (c) showed the necrotic area (black arrows) and residual tumor (red arrows) which was positive at CD34 staining (d).

**Figure 8:**
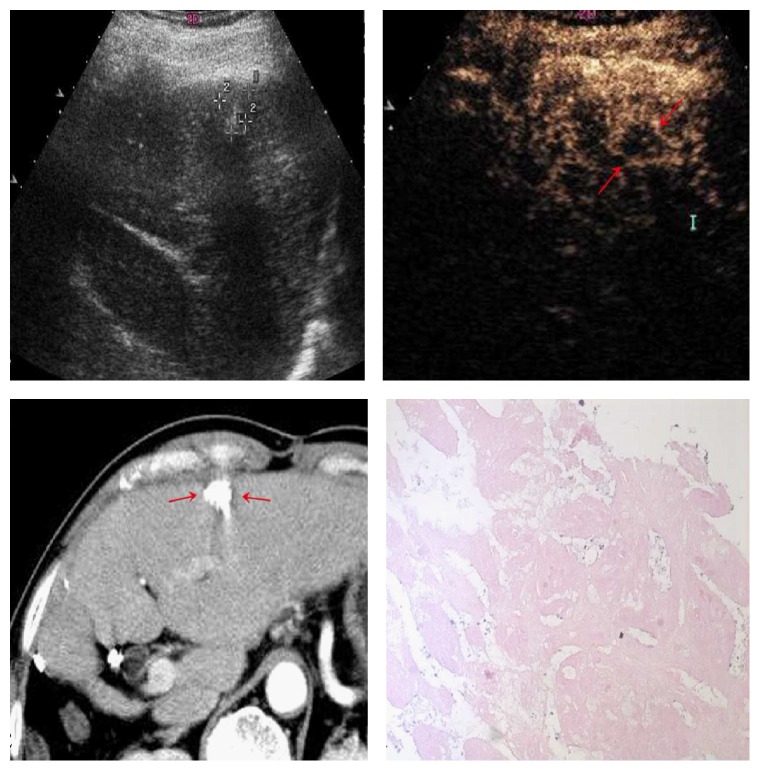
35-year-old man with 1.6 cm×1.3 cm lesion (cross mark). CEUS images in the arterial phase (a, b) showed peripheral rim-like arterial phase enhancement (red arrows), probably representing reactive hyperemia, while the CT examination showed complete lack of enhancement (c) (red arrows). Pathological results showed complete necrosis of the tumor (d).

In group 1, 38 residual lesions (88.4%) were hyperenhanced and the other 5 were iso-enhanced in the arterial phase. The enhanced pattern was inhomogeneous (nodular, branch, etc.) in 11, homogeneous in 11, partial in 12, peripheral in 7, and peripheral rim-like in 2 (Figures 1–8). The longest tumor sizes in each type in group 1 are shown in Table 3. The iso-enhanced pattern was showed in 5 of the 11 homogeneous lesions. In group 2, 28 of all the lesions were hyperenhanced in the arterial phase.

### 3.3. Portal Venous Phase and Late Phase

In group 1, 30 residual lesions (69.8%) were iso-enhanced and 13 were hypoenhanced in the portal venous phase. 27(62.8%) lesions stayed iso-enhanced in the late phase and showed a clean-out after 4 min. And the rest of 16 lesions were hypoenhanced in the late phase. In group 2, 5 of well-differentiated lesions were iso-enhanced in the portal venous phase, with complete washout more than 4 min later. The rest 25 (83.3%) lesions were hypoenhanced through the portal venous phase.

## 4. Discussion

Minimally invasive conventional treatments such as TACE, radiofrequency ablation, and percutaneous ethanol injection have been widely used with favorable results for inoperable HCC. However, incomplete tumor necrosis and frequent recurrences limit the usefulness of these techniques in some patients, particularly those with intratumoural vascularity, multimodal type of HCC, or portal vein thrombosis [9, 10]. As a result, patients receiving these treatments need to be monitored to assess tumor response and to plan further treatment for residual tumor.

Pathologically, HCCs develop in a multistep process in patients with liver cirrhosis. This process includes progression from low-grade dysplastic nodules to high-grade dysplastic nodules and finally to overt HCC [11]. These morphologic changes are paralleled by hemodynamic changes including the loss of portal tracts and gradual development of new arterial vessels that become the dominant blood supply in overt HCC lesions. This arterial neoangiogenesis is a key factor in the imaging diagnosis of HCC. According to the latest AASLD guidelines, HCC is typically characterized by arterial hypervascularity of washout during the portal venous phase. Several reports have demonstrated that in 90% of HCC and particularly in higher grade HCC, arterial enhancement showed washout in the portal and/or late phase (*p*< 0.0001) [12–15]. In our study, 25(83.3%) of group 2 lesions showed this typical enhancement pattern (quick washin and quick washout). 28 of our well-differentiated HCCs showed hypervascularity in the arterial phase.

In our study, not all of the residual tumors were available for percutaneous needle biopsy, and some of them were confirmed by the MRI results and postoperative pathologic results. Wakasa [16] reported that small HCC (maximum diameter: l.1-2.0 cm), even when encapsulated, frequently invade blood vessels and adjacent liver tissue. We surmise that the peripheral enhancement we observed may be caused by satellite lesions which are nourished by a collateral artery or the progression of intracapsular invasion. With regard to the tumors with partial and inhomogeneous (nodular, branching) hyperenhanced residual tumors, the investigations of Sakurai [17] and Hsu [18] both suggested that residual tumor after treatment occurred mainly at the tumor periphery, beneath the tumor capsule, or around the fibrous septa in the larger tumors (largest diameter, 6.5-15 cm). Moreover, partial recanalization of the embolized artery may contribute to the regrowth of the residual tumor [17]. This may explain the formation of some homogeneous hyperenhancing residual tumors. Due to blood supply from the portal vein after hepatic artery occlusion, 5 lesions appeared homogeneous iso-enhanced in the arterial phase. If both of the arterial and portal blood supplies were blocked, the lesion would be completely necrotized, but this is not feasible clinically. In these particular cases, other interventional therapies should be considered to achieve a complete response. Studies show that combinations of different nonsurgical methods may be effective in improving the survival rate [19].

What kind of nonsurgical therapy should be considered for further treatment? In our opinion, in addition to the enhancement pattern, enhancement kinetics also should be taken into consideration. Results of studies on posttherapeutic parametric evaluation with CEUS for TACE have not reached an agreement [20, 21]. In our study, there was no significant change in the rise time or peak time after TACE. Pei et al. [9] also reported that the number of unpaired arteries (UAs) had moderate correlation with RT and PT and that the WT was associated with output perfusion velocity. A significant increase in peak time which suggested an effective vessel blocking was associated with good response after TACE [22]. In the investigation of Moschouris [21], they attributed this to the small number of patients studied. In our study, this could be contributed to that most of the hyperenhanced residual tumor (88.4%) in arterial phase. This did not mean the failure of TACE treatment, because we studied only the residual tumor not the whole one, and further treatment to these residual tumors could achieve good response as well. For rise time the systemic error of bolus injection speed also could have an influence on its results. Moreover, because of various numbers and sizes of the tumor with abnormal blood vessels, some but not all the researchers found statistically significant correlations between histological angiogenesis and enhancement parameters under different sample treatments [22, 23]. So this could also give explanations to the peak time in group 1. Our results showed that the washout time was increased after TACE (P<0.05). As what we have mentioned above, the slower the enhancement signal disappears (i.e., slow washout time), the richer the feeding vessels branch from portal tracts is. Twenty-seven residual tumors (62.8%) in group 1 showed complete washout after 4 min. For inhomogeneously and hypoenhanced residual tumors, hepatectomy, radiofrequency ablation, or percutaneous ethanol injection would be selective methods for further treatment. For some partial and homogeneously enhanced nodules showing hyperenhancement in the arterial phase but slow washout due to tumor blood supply from the portal vein, additional cycles of TACE most likely would not result in improved outcomes. In group 1, for the hyperenhanced lesions that were larger than 3 cm and lesions which showed typical HCC washout in portal venous phase, an additional treatment with TACE may be indicated if the digital subtraction angiography examination shows a favorable result. Otherwise, other interventional therapies would be preferred, alone or in combination.

Our study has certain limitations. First, this is a single center study with limited sample size. Second, we have not collected data about preembolization CEUS behaviour of the tumor, which could be a potential confounding factor.

## 5. Conclusions

The perfusion patterns of residual lesions on CEUS after TACE differed from those of untreated HCCs. In particular, longer washout times in some treated lesions indicate that some residual tumors recruited a new blood supply from the portal venous system after the arterial blood supply was blocked. In these patients, more targeted interventional therapies, such as radiofrequency ablation, percutaneous ethanol injection may be more effective than further treatment with TACE.

## Figures and Tables

**Table 1:** Clinical characteristics of patients in this study.

Clinical data	Number (%) (group 1/group 2)	Mean ±SD (range) (group 1/group 2)	*t*/*χ*^2^	*p*
Patients				
Female	3 (7.0)/3 (10.0)			0.69^*τ*^
Male	40 (93.0)/27 (90.0)			

Age (years)				
group 1		56.1±7.9 (24-82)	1.51	0.07
group 2		53.9±1.1 (34-77)		

HBs-Ag				
positive	43 (100.0)/ 28 (93.3)			0.166^*τ*^
negative	0 (0)/2 (6.7)			

Number of lesions per patient (group 1/group 2)				
1	34 (79.1)/20 (66.7)		1.82	0.402
2	5 (11.6)/7 (23.3)			
>2	4 (9.3)/3 (10.0)			

The longest diameter of single HCC (cm)				
<2	10 (23.3)/ 24 (80.0)	1.8±0.3/1.5±0.6	1.5	0.07
2–5	19 (44.2)/ 4 (13.3)	2.9±0.9/3.3±0.7	-0.83	0.21
>5	14 (32.5)/ 2 (6.7)	6.0±0.4/5.3±1.5	6.31	0.32

Aetiology of liver disease (group 1/group 2)				
Alcoholic	0 (0)/2 (6.7)			0.166^*τ*^
Hepatitis B	43 (100.0)/28 (93.3)			

Differentiation of HCC (group 1/group 2)				
Well-differentiation	21 (48.8)/12 (40)		0.754	0.385
Moderate-poorly differentiation	22 (51.2)/18 (60)			

^*τ*^: Fisher's exact test.

**Table 2:** Perfusion characteristics of the hepatocellular carcinomas on CEUS.

Perfusion	Number (%)	Mean±SD (sec)	*t*/*χ*^2^	*p*
Rise Time				
group 1		16.1±2.7	1.38	0.09
group 2		15.1±3.5		

Time to Peak				
group 1		31.3±3.1	0.54	0.30
group 2		30.9± 3.2		

Washout Time				
group 1		191.0± 31.3	7.77	<0.01
<4min	16 (37.2%)			
>4min	27 (62.8%)			
group 2		142.6± 16.1		
<4min	25 (83.3%)			
>4min	5 (16.7%)			

Arterial Phase (group 1/group 2)				
Hyper-vascularity	38 (88.4%)/28 (93.3%)		0.502	0.479
Iso-vascularity	5 (11.6%)/2 (6.7%)			

Portal Venous Phase (group 1/group 2)				
Iso-enhanced	30 (69.8%)/5 (16.7%)		19.965	<0.01
Hypo-enhanced	13 (30.2%)/25 (83.3%)			

Late phase (group 1/group 2)				
Iso-enhanced	27 (62.8%)/5 (16.7%)		15.27	<0.01
Hypo-enhanced	16 (37.2%)/25 (83.3%)			

**Table 3:** Comparison between the five enhancement patterns in group 1.

No.	Enhancement Pattern	Patient Number	Tumor Size (Mean ±SD)	*t*	*p*
1	Inhomogeneous	11	4.7±1.2		
2	Homogeneous	11	2.9±1.0	3.82	<0.01
3	Partial	12	3.1±1.7	2.58	<0.01
4	Peripheral	7	2.5±0.6	4.47	<0.01
5	Peripheral rim-like	2	2.1±0.4	5.66	<0.01

## Data Availability

The data used to support the findings of this study are available from the corresponding author upon request.

## Abbreviations

CEUS: Contrast-enhanced ultrasound
TACE: Transarterial chemoembolization
HCC: Hepatocellular carcinoma
RFA: Radiofrequency ablation
PEI: Percutaneous ethanol injection
MRI: Magnetic resonance imaging
CT: Computed Tomography
RT: Rise time
PT: Peak time
WT: Washout time.
